# Identifying Dietary Timing of Organic Trace Minerals to Reduce the Incidence of Osteomyelitis Lameness in Broiler Chickens Using the Aerosol Transmission Model

**DOI:** 10.3390/ani14111526

**Published:** 2024-05-22

**Authors:** Khawla Alharbi, Andi Asnayanti, Anh Dang Trieu Do, Ruvindu Perera, Layla Al-Mitib, Abdulkarim Shwani, Marco A. Rebollo, Michael T. Kidd, Adnan Ali Khalaf Alrubaye

**Affiliations:** 1 Cell and Molecular Biology Program, University of Arkansas, Fayetteville, AR 72701, USA; ka030@uark.edu (K.A.); aasnayan@uark.edu (A.A.); ad086@uark.edu (A.D.T.D.); rperera@uark.edu (R.P.); lhalmiti@uark.edu (L.A.-M.); 2 Center of Excellence for Poultry Science, University of Arkansas, Fayetteville, AR 72701, USA; mkidd@uark.edu; 3 National Agency of Drug and Food Control, Jakarta 10520, Indonesia; 4 Southeast Poultry Research Laboratory, U.S. National Poultry Research Center, ARS, USDA, Athens, GA 30605, USA; abdulkarim.shwani@usda.gov; 5 Zinpro Corporation, Eden Prairie, MN 55344, USA; mrebollo@zinpro.com

**Keywords:** broiler, bone, lameness, chondronecrosis, organic trace minerals

## Abstract

**Simple Summary:**

Lameness induced by bacterial chondronecrosis with osteomyelitis poses a significant challenge in the poultry industry globally. It is caused by pathogenic bacteria entering the bird’s bloodstream via the skin, respiratory tract, or digestive system. These bacteria are then disseminated hematogenously and infest the osteochondrotic crypts from chronic fractures resulting from mechanical stress caused by fast growth rate and heavy body weight, leading to lameness. The objective of this research is to identify the ideal timing for Availa^®^ ZMC supplementation in broiler lameness reduction using an aerosol transmission challenge model, with a hypothesis that providing Availa^®^ ZMC through the initial four weeks of the production cycle will optimally strengthen skeletal bones, improve intestinal integrity, and enhance immune responses. The results show that including 0.15% Availa^®^ ZMC in the broiler diet during the first 28 days is the optimal timing. This approach provides a level of protection against BCO lameness nearly equivalent to that of birds fed with the same inclusion level throughout the entire production cycle.

**Abstract:**

Our prior research demonstrated a 20% to 25% reduction in bacterial chondronecrosis with osteomyelitis (BCO) lameness in broilers with organic Zn, Mn, and Cu (Availa^®^ ZMC) supplementation. Expanding on this, we investigated the optimal timing for Availa^®^ ZMC feeding to mitigate BCO lameness and reduce feed additive costs in the poultry industry. In this study, we compared the application of 0.15% Availa^®^ ZMC for 56 days, the first 28 days, and the last 28 days. The experimental design was a randomized block design involving 1560 one-day-old chicks distributed across two wire-floor pens as BCO source infection and four treatment groups with six replicates. The source of BCO infection exhibited a cumulative lameness incidence of 83%, whereas the negative control group showed a 77% cumulative incidence of lameness (*p* = 0.125). Administering 0.15% of Availa^®^ ZMC during the initial 28 d resulted in a 41.3% reduction in BCO incidence, significantly different from the supplementation during the last 28 d (*p* < 0.05). However, this reduction did not differ substantially (*p* > 0.05) from the 56d application period. Hence, administering 0.15% Availa^®^ ZMC during the first four weeks emerges as the optimal timing protocol, providing a defense against lameness comparable to the continuous supplementation throughout the complete production duration. Implementing this feeding approach reduces the cost of feed additive, promotes the health of skeletal bones, and effectively protects against BCO lameness in broilers, offering a valuable consideration for producers seeking optimal outcomes in the poultry industry.

## 1. Introduction

Lameness or leg weakness is a prevalent skeletal disease affecting broilers globally, leading to substandard well-being and substantial financial losses due to increased feed conversion ratio (FCR), decreased productivity, increased mortality rate, and culling [1,2]. Lameness is associated with a complex etiology that includes various factors such as genetic traits, contagious agents, body structure, activity levels, substandard litter condition, stocking density, and diet [3,4,5,6,7,8]. Bacterial chondronecrosis with osteomyelitis (BCO) is the predominant trigger of lameness in rapidly growing broilers, resulting in significant animal well-being concerns and economic losses [9,10,11]. BCO is triggered by the imbalance between body mass growth and skeletal maturation, which is regarded as the key factor determining BCO susceptibility in the broiler industry [1,3,12]. BCO in broilers leads to productivity and financial losses because of broiler condemnation during the marketing lifetime [2,13], as well as animal welfare issues because of pain associated with leg bone disorders [14] and limited ability to access water and feed [15]. BCO lameness pathogenesis in chickens entails bacterial contagions, intestinal permeability, high septicemia, joint arthritis, and eventually bone necrosis on the proximal epiphyseal plates of the femora and tibiae [2,9,11,12,16,17]. BCO lameness typically manifests in birds aged between 14 and 70 days old [1]. Our research identified *Staphylococcus agnetis* as one of the main bacteria linked to BCO lameness in our research barn [10,16,18]. Additionally, *Escherichia coli*, *Enterococcus cecorum*, and *Staphylococcus hyicus* were isolated from commercial farms and are closely related to *S. agnetis* [10,18,19]. These bacteria typically move from the respiratory and intestinal systems into the blood circulatory system and are distributed throughout the body. Making matters worse, low hygiene and high density of broiler production in commercial farms further aggravate the progression of BCO lameness disease.

Trace minerals (TMs) constitute a small fraction of the diet yet are critical in maintaining the health and productivity of broiler chickens. TMs function as essential co-factors of metalloenzymes involved in diverse metabolic pathways, hormone secretions, connective tissue synthesis, and immune systems [20,21,22,23,24,25]. Specifically, TMs such as Zn, Mn, and Cu, are important for bone development and immune systems in poultry production. Zinc stimulates collagen synthesis and turnover of bone cartilage, promotes body weight gain, and optimizes cellular and humoral immunity [26,27,28,29], while Cu acts as a collagen–elastin bridge [30,31]. An adequate Cu concentration in the diet promotes optimum bone mass accretion in the long bones and strengthens femoral bones [32,33]. On the other hand, copper deficiency disrupts bone mineralization in birds [30], while its excessive intake interferes with mineral absorption, particularly Mg, for bone matrix formation [34]. Finally, Mn is crucial for the growth of the long bone, tendon, and tibiometatarsal joints of broilers [35]. Manganese stimulates an immune response through its interaction with heterophils and macrophages [36] and increases the activity of natural killer cells [37]. Therefore, TMs highly affect the bone development, growth performance, and immune functions of broiler chickens.

Although TMs play an essential role in poultry nutrition, most feed ingredients do not contain a proper proportion of TMs required for animal growth and bone development [22,38,39]. Mineral deficiency in poultry diet leads to reduced feed intake, low immune defense, and thereby low production [35]. Traditionally, TMs in commercial poultry diet premixes were provided in the form of inorganic salts, including oxides, sulfate, or carbonates. However, irregular amounts of such elements have been shown to result in antagonistic effects and low bioavailability [40]. Inorganic minerals from salts are also implicated in environmental pollution because of mineral accumulation from poultry waste [38]. Therefore, organic mineral sources in the form of complexed or chelated minerals have been identified as an alternative for poultry diets due to their higher bioavailability [40]. Some studies also reported that birds fed organic minerals (e.g., Zn, Cu, Mn, and Fe) gained more body weight and improved feed conversion compared to birds fed diets with inorganic minerals [41,42,43].

Considering the benefits of organic TM-supplemented diets on poultry bone health and immune defense, we thus investigated the effect of organic TM combinations (mineral amino acid complex) on BCO lameness occurrence in broilers. Previously, we tested the efficacy of the commercially available supplement, Zinpro^®^ Availa^®^ ZMC, which contains the essential trace minerals Zn, Mn, and Cu, in reducing broiler BCO lameness. The results indicated that Availa^®^ ZMC decreased BCO lameness incidence by 20–25% by enhancing the integrity of the tight junctions in the intestinal barrier and promoting the antibacterial activity of monocytes [11]. However, supplementing broiler feed with a commercial organic trace mineral for the entire production cycle would potentially entail significantly higher costs associated with feed additives. Thus, we extended our investigation to determine the optimal timing for Availa^®^ ZMC supplementation with a hypothesis that providing Availa^®^ ZMC during the first 4 weeks of the production period would optimally strengthen skeletal bones, improve intestinal integrity, and enhance immune responses. Thus, this feeding strategy is expected to protect broilers from BCO lameness while reducing the cost of feed additives for the broiler industry.

## 2. Materials and Methods

### 2.1. Animal Research and Facility

This study was conducted at the University of Arkansas Research Farm between 15 December 2022 and 10 February 2023. A total of 1560 one-day-old male Cobb 500 chicks were distributed across 26 pens, with each measuring 1.5 m × 3.0 m and equipped with either suspended wire flooring or wood shaving flooring. The density was initially set at 60 chicks per pen on day 0, which was reduced to 50 birds per pen by day 14 to maintain the ideal stocking density. Two wire-floor pens for the BCO source group and six pens were assigned to each of the four treatment groups as treatment replicates in this study.

The research facility was outfitted with photoperiod regulation set to a 23 h light to 1 h dark ratio for the entirety of this study. The temperature schedule was programmed at 32 °C for days 1 to 3, 31 °C for days 4 to 6, 29 °C for days 7 to 10, 26 °C for days 11 to 14, and 24 °C thereafter. Tunnel ventilation, overhead space heaters, and cooling pads were installed in the research house for automatic environmental control. Each pen was equipped with a single water line at one end and two feeders at the opposite end. All water pipelines were decontaminated using 10% bleach and flushed with fresh water before the experiment.

### 2.2. Experimental Design

This study includes a BCO source group in wire-floor pens receiving Diet A (basal diet) for 56 days and four dietary treatments for chicks reared in litter-floor pens: a negative control group received Diet A (basal diet) for 56 days, a second group was fed Diet B containing 0.15% Availa^®^ ZMC throughout the 56 days, a third group (Diet C) received Diet A for the first four weeks (1–28 d) and switched to Diet B for the last four weeks (29–56 d), and a fourth group (Diet D) received Diet B for the first four weeks (1–28 d) and switched to Diet A for the last four weeks (29–56 d) (Table 1). The Availa^®^ ZMC treatment is manufactured by Zinpro Corporation, Eden Prairie, MN, USA. Availa^®^ ZMC is a mix of Zn, Mn, and Cu amino acid complexes and contains the following rates of each mineral: Zn 4.0%, Mn 4.0%, Cu 0.7% bound into 1-to-1 amino acid ligand. The amino acid contained additions of up to 25.1% crude protein. In this study, we added 1.5 g of Availa ZMC^®^ per kg of feed provided 60 ppm of Zn, 60 ppm of Mn, and 10.5 ppm of Cu complexed to an amino acid.

The feed formulation specified in Table A1 was created at the Poultry Research Feed Mill at the University of Arkansas to meet commercial specifications (Cobb feeding guide). All chickens had access to clean ad libitum water and feed in the form of a starter crumble feed from ages 0 to 34 days and a finisher pellet feed from ages 35 to 56 days.

### 2.3. The BCO Challenge Model

The impact of dietary treatment on BCO lameness was evaluated by employing an aerosol transmission model [44]. This model combines wire-floor pens and wood shaving flooring pens. The two wire-floor pens were used as a source group for BCO or seeder birds. Every pen was equipped with a single water pipeline and two feeders placed on opposite ends to encourage the birds to move back and forth to access both water and feed. The wire floors were positioned upwind of the four dietary treatment groups raised in wood shaving litter pens. This setup facilitates the dissemination of BCO from the wire to the litter flooring pens. The outbreak of BCO on the wire flooring is spread to the litter floors via the air circulated by fans located at the rear of the facility. Buffer zones (empty areas) were established between the BCO source and dietary treatment groups to avoid direct contact between the seeder birds and those receiving treatment.

### 2.4. Lameness Assessment

The lameness trial was run for 56 d and daily lameness assessment was conducted from day 22 until day 56 of age. The birds were prompted to move to diagnose symptoms of lameness, such as immobilization, abnormal gait, and spinal deformity (kinky back), and were recorded as clinically lame. After diagnosis, the birds were humanely euthanized and necropsied for evaluation of the tibial and femoral heads of BCO lesion progressions. The femoral and tibial lesion developments were recorded and classified by Wideman, 2016 [2]: N = femur head and proximal tibia appear entirely normal; FHS = proximal femoral head separation (epiphyseolysis); FHT = proximal femoral head transitional degeneration; FHN = proximal femoral head necrosis; THN = proximal tibial head necrosis; THNC = proximal tibial head necrosis caseous; THNS = proximal tibial head necrosis severe; TD = tibial dyschondroplasia; KB = kinky back (spondylolisthesis); SDS = sudden death syndrome (flipover, heart attacks); DUR = death due to unknown reasons. Total cumulative lameness = FHS + FHT + FHN + THN + THNS + THNC + TD + KB.

### 2.5. Bacterial Identification

Bacteria isolated from BCO lesions were grown and further identified by using DNA sequencing of the rDNA 16S V1-V5 region according to the protocol of Asnayanti et al., 2024 and Alharbi et al., 2024 [45,46].

### 2.6. Data Analyses

The lameness data were inputted and analyzed using Microsoft Excel 2018 (Microsoft, Redmond, WA, USA) and the cumulative lameness percentage was calculated by the formula of the total number of lame birds for treatment divided by the total number of birds in the treatment group on D14 and multiplied by 100. The impact of dietary treatments was analyzed through logistic regression with a binomial distribution, utilizing the generalized linear model (GLM) procedure in R version 4.2.2 (R Foundation for Statistical Computing, Vienna, Austria). All statistical significance was established at *p* < 0.05.

## 3. Results

**Figure 1:**
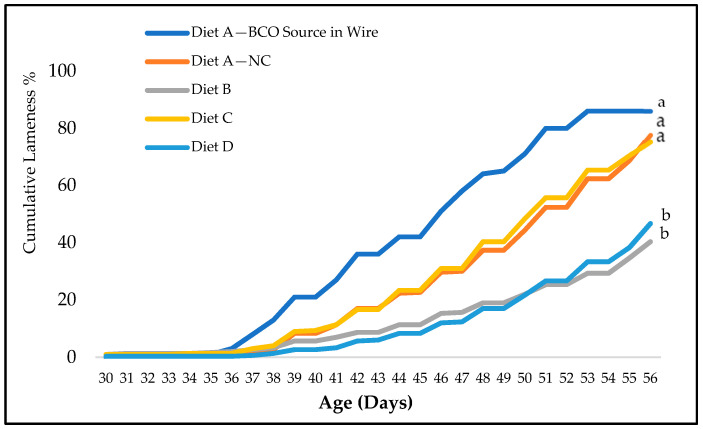
The percentage of the cumulative lameness data in BCO source in wire group and four treatment groups from day 30 to day 56. Diet A: basal diet for 56 days, Diet B: 15% Availa^®^ ZMC for 56 days, Diet C: Diet A on the first four weeks (1–28 d) switched to Diet B on the last four weeks (29–56 d), Diet D: Diet B on the first four weeks (1–28 d) switched to Diet A on the last four weeks (29–56 d). ^a,b^ Values within a class with different letters vary significantly at *p* < 0.05.

Birds on wire flooring exhibited a notable rise in lameness over time, reaching a cumulative rate of 83.0%. The infection was effectively spread to the negative control group of birds housed on litter flooring, resulting in a cumulative lameness rate of 76.3% (*p* = 0.12). Conversely, birds fed a diet with 15% Availa^®^ ZMC for the first 28 days (Diet D) and for 56 days (Diet B) exhibited the lowest cumulative lameness rates of 45% and 37%, respectively (*p* < 0.05). BCO lameness first emerged in the source infection group at 34 d of age, where it subsequently began to spread to other treatment groups on d 37 (Figure 1).

**Figure 2:**
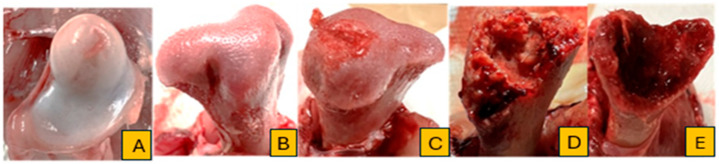
The classifications of femoral head lesions for BCO lesions developments: (**A**) = normal; (**B**) = femoral head separation; (**C**) = femoral head transitional degeneration; (**D**,**E**) = femoral head necrosis.

**Figure 3:**
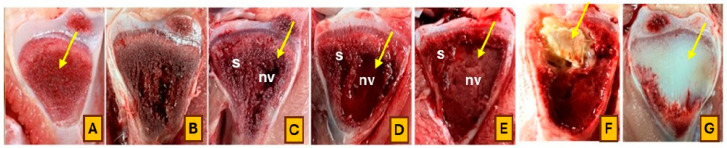
The classifications of tibial head lesions for BCO lesions developments: (**A**) = normal; (**B**) = tibial head necrosis; (**C**–**E**) = tibial head necrosis severe; (**F**) = tibial head necrosis caseous; (**G**) = tibial dyschondroplasia.

As shown in Figure 2 and Figure 3, we conducted a macroscopic evaluation to identify the severity of BCO lesions on both sides of the proximal head of the femur and tibia. A normal femoral head (N) appeared to be a complete epiphyseal articular cartilage enveloping the proximal epiphyseal plate, as depicted in Figure 2A. The next lesion category is proximal femoral head separation or epiphyseolysis (FHS), in which the surface of the proximal femoral epiphysis has separated from the cartilage, and an undamaged appearance remains within the acetabulum, as shown in Figure 2B. The subsequent category, proximal femoral head transitional degeneration (FHT), displays apparent lesions with varying levels of damage in the growth plate cartilage, as illustrated in Figure 2C. The most acute damage lesion is femoral head necrosis (FHN), which manifests as a fracture, perforation, and necrosis of the weakened proximal femoral epiphysis and physis upon the femur’s dislocation, leading to the loss of articular cartilage over the proximal epiphyseal plate, as shown in Figure 2D,E.

Additionally, Figure 3 depicts tibial head necrosis development levels leading to BCO lameness. A normal proximal tibial head with trabecular bone stents in the metaphyseal area is presented in Figure 3A, providing essential support to the growth plate. Figure 3B–F reveal signs of bone damage due to osteomyelitis, comprising bacterial leaking, necrotic voids (nv), sequestra (s), and small fractures beneath the growth plate (indicated by arrows). Furthermore, Figure 3G illustrates tibial dyschondroplasia, characterized by abnormal clusters of cartilage in place of cancellous bone in the proximal metaphyseal area of the tibiotarsus.

**Figure 4:**
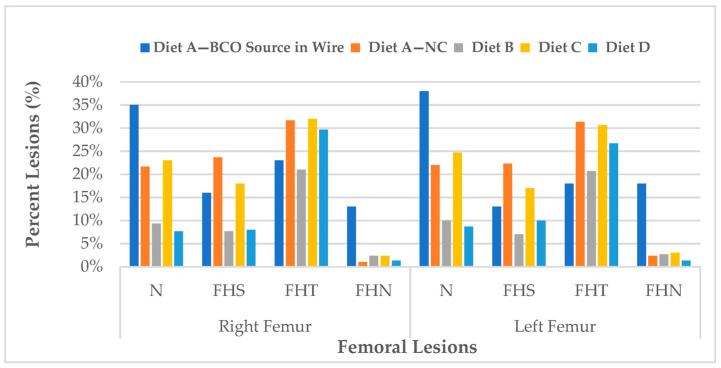

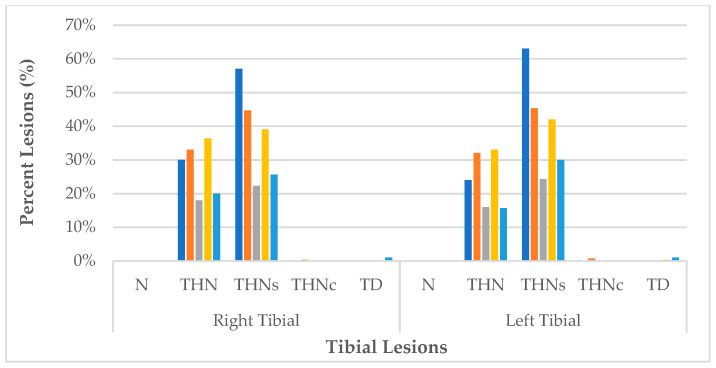
The cumulative percentage of diagnoses for femoral lesions (**top**) and tibial lesions (**bottom**) in all lame broilers in each treatment. Diet A: basal diet for 56 days, Diet B: 15% Availa^®^ ZMC for 56 days, Diet C: Diet A on the first four weeks (1–28 d) switched to Diet B on the last four weeks (29–56 d), Diet D: Diet B on the first four weeks (1–28 d) switched to Diet A on the last four weeks (29–56 d).

Figure 4 displays the ratio of cumulative lameness lesions in both legs of broilers infected with BCO, with the data categorized by the tibia and femur. Generally, THNS in the tibia and FHT in the femur is one of the main frequently identified categorized lesions across all treatments, with no distinguishable types in BCO lesion patterns observed between the right and left legs. The inclusion of 0.15% Availa^®^ ZMC in the diet for the last 28 days resulted in the highest incidence of FHT and THNS lesions in the femur and tibia bones, at 30% and 40%, respectively. There was no significant variation compared to the BCO source and negative control groups (*p* > 0.05).

Table 2 illustrates the effects of treatment groups on BCO in the last three weeks on days 42, 49, and 56. Significantly, the BCO source group showed the highest lameness rates on days 42, 49, and 56, with prevalences of 36%, 65%, and 83%, respectively. Meanwhile, the negative control group and the Diet 1–2 group exhibited the second-highest lameness rates on these days, with prevalences of (19%, 38%, and 76%) and (14%, 40%, and 76%), respectively. Over the period, all treatment groups experienced an average weekly rise in lameness varying from approximately 10% to 40%.

**Figure 5:**
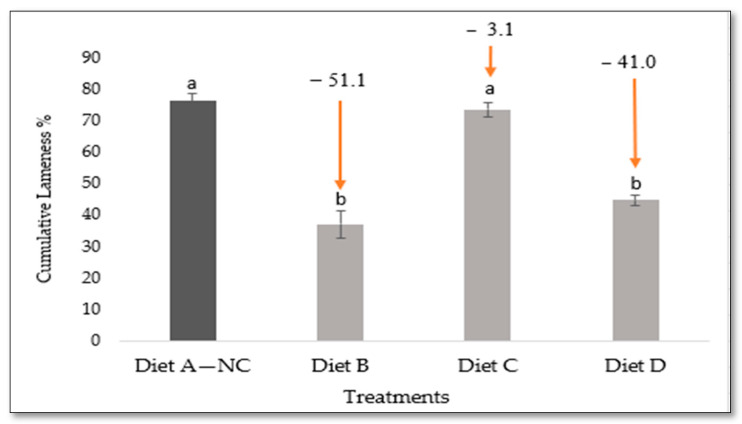
Comparing the effectiveness of dietary treatments for lameness reduction for identifying the ideal timing of Availa^®^ ZMC application (Diet B, Diet C, and Diet D) by comparing them with Diet A—negative control (NC). ^a,b^ Values with distinct superscripts in the same category are significantly different at *p* < 0.05.

Administering Availa^®^ ZMC to broilers throughout the full 56 days and the initial 28 days reduced the occurrences of lameness compared to the negative control by 51.1% (37.3% vs. 76.3%, *p* < 0.05) and 41.3% (45.0% vs. 76.3%, *p* < 0.05), respectively (Figure 5).

**Figure 6:**
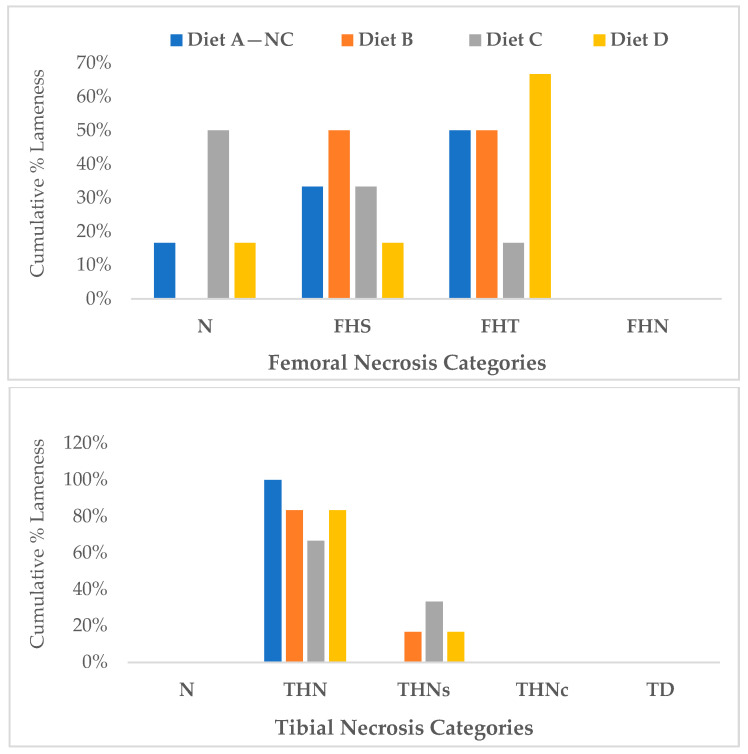
Proximal femoral and tibial head lesions of apparently healthy chickens on day 56. The severity categories for tibial and femoral lesions and their incidence rates, according to Wideman, 2016 [2]: normal femoral and tibial head (N); femoral head separation (FHS); femoral head transitional lesions (FHT); femoral head necrosis (FHN); tibial head necrosis (THN); tibial head necrosis severe (THNS); tibial head necrosis caseous (THNC); tibial dyschondroplasia (TD). Diet A: basal diet for 56 days, Diet B: 15% Availa^®^ ZMC for 56 days, Diet C: Diet A on the first four weeks (1–28 d) switched to Diet B on the last four weeks (29–56 d), Diet D: Diet B on the first four weeks (1–28 d) switched to Diet A on the last four weeks (29–56 d).

At 56 days, five birds from each treatment group that exhibited no signs of lameness (seemingly healthy) were necropsied to evaluate femoral and tibial health. Notably, these birds presented with BCO lesions, with FHT and THN being the predominant categories for femoral and tibial lesions (Figure 6).

**Figure 7:**
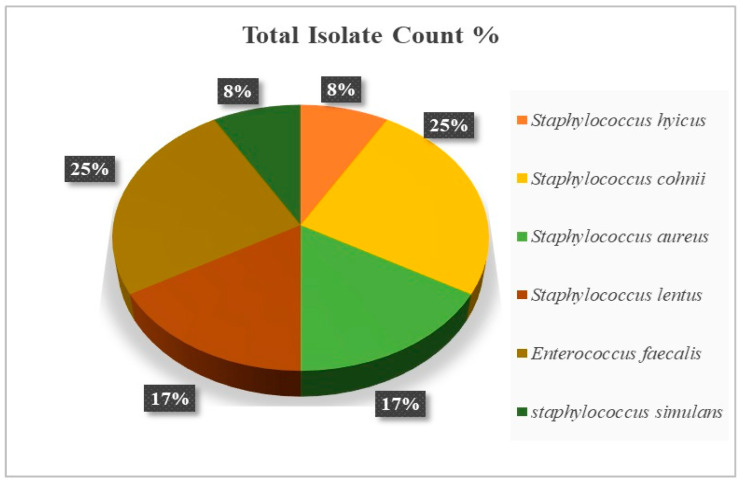
Identification of bacterial strains in the proximal femural and tibial heads in apparently healthy broilers across all treatment groups on day 56.

Moreover, the characterization of bacterial species in BCO lesions shows a prevalence of 8% for *Staphylococcus hyicus*, 25% for *Staphylococcus cohnii*, 17% for *Staphylococcus aureus*, 17% for *Staphylococcus lentus*, 8% for *Staphylococcus simulans*, and 25% for *Enterococcus faecalis*, as illustrated in Figure 7.

## 4. Discussion

This research was performed to evaluate the optimum timing of applying Availa^®^ ZMC supplementation to mitigate the occurrence of bacterial chondronecrosis with osteomyelitis in broilers using an aerosol transmission model. This model was utilized to induce BCO, in which birds were allocated to two suspended wire-floor pens designated as the BCO source group. Our findings demonstrated the effectiveness of the experimental model in inducing lameness starting at 34 days, which subsequently spread to groups of birds residing on litter floors. The BCO progression within the source group corresponds with an earlier investigation that identified a significant occurrence of lameness in birds raised on a wire-floor pen after reaching 35 d of age, which eventually spread to other treatment groups after 3 to 5 days [9,11,44,45,46]. The wire flooring utilized in the aerosol transmission model generates shear stress on the leg skeletons of broilers as they move to access water and feed, which are placed in opposite directions within the coops. Broilers’ fast weight gain intensifies their leg weakness on wire floors, facilitating the movement of pathogens from the pulmonary and digestive tracts into the blood circulation by weakening the tight junction intestinal barrier [2,9,11,17,44,45,46]. As a result, the causative agents of BCO were dispersed and traveled from the infected source coops to the unexposed broilers in the litter flooring coops at the same facility, carried by an air circulation gradient generated by the exhaust fans.

The results of BCO lesions from various treatments are represented by the progression of femur and tibia disorders (Figure 2 and Figure 3). FHN is the most severe lesion in the femoral head, distinguished by perforation, fracturing, and necrosis [47]. This case leads to losing the articular cartilage that normally caps the growth plate, resulting in birds suffering from FHN exhibiting severe paralysis symptoms, leaving them completely immobilized and unable to move [14]. This paralysis leads to restricting the access to food and water, resulting in acute dehydration and death [2,46,48]. Conversely, broilers with FHT and FHS exhibit moderate lesions. Typically, the surfaces appear fairly undamaged and smooth, with gross lesions visible from the wound in the epiphyseal plate. Although these lesions are less severe, they affect the broilers’ ability to stand and uphold equilibrium, leading to a hesitation to move and an abnormal gait. The progression of tibial lesion severity reflected the levels of femoral head damage, characterized by a developing necrotic area that reaches toward the growth plate of the proximal tibial head, transitioning from tibial head necrosis (THN) to tibial head necrosis severe (THNS) [2].

The results implied that broilers subjected to Availa^®^ ZMC treatment experienced protection from lameness disease until they reached 42 d old (Table 2). These data, consistent with prior studies, indicate that adequate absorption and deposition of minerals, particularly calcium, phosphorus, manganese, zinc, and vitamin D3, play a crucial role in forming the bone matrix during the early life of broilers, significantly affecting bone and intestinal health [45,49,50,51]. Therefore, our results support the hypothesis that incorporating 0.15% Availa^®^ ZMC effectively reduces the occurrence of lameness in chickens up to 42 days old, yet further research is required to clarify this finding.

Remarkably, the results in Figure 6 show that BCO lesions are present in apparently healthy birds without any symptoms of lameness at day 56. This finding is supported by prior studies indicating that BCO disease progresses even in seemingly healthy birds at day 56 and is prevalent in broiler chickens [45]. The bacteria isolated from the lesions in the proximal tibia and femoral head of sub-clinically healthy birds comprised 75% *Staphylococcus* spp. of the total identified bacteria. This finding aligns with prior investigations suggesting that BCO lameness is attributable to the infection by a diverse group of opportunistic organisms, with *Staphylococcus* spp. as one of the most frequently encountered species in broilers with lameness at our primary research facilities [12,45,48,49,50,51]. Consequently, this result indicates that BCO disease has progressively manifested in seemingly healthy broilers by day 56, which supports the practical advice to avoid extending the production cycle of broilers beyond 56 days.

The poultry industry is constantly prioritizing the reduction of feed production costs due to many challenges, including the increasing cost of traditional feed ingredients and the need to increase animal productivity and reduce production costs. Using organic zinc, manganese, and copper in conjunction has been recognized as a practical approach to improving poultry health, minimizing bacterial pathogen colonization, and reducing femoral head necrosis [23,24,25,26,27,28,29,30,31]. Thus, in this study, we identified the best timing for inclusion of Availa^®^ ZMC to control BCO lameness and minimize feed additive costs in the poultry industry. We indicated that the inclusion of Availa^®^ ZMC supplementation in the broiler diet for the initial four weeks has been found to confer protection against lameness comparable to that offered over the entire production cycle. This intervention represents a feasible and practical way to manage lameness in broiler chickens. Furthermore, this strategy has the potential to efficiently reduce feed additive and production costs within the poultry industry, aligning with the objectives of numerous farm producers. Further research is needed to more accurately establish the maximal production lifespan of broilers supplemented with Availa^®^ ZMC while preserving BCO resistance characteristics, thus helping to achieve optimal production efficiency and improve broiler health and welfare at the same time.

## 5. Conclusions

This study assessed the impact of administering 0.15% Availa^®^ ZMC over different periods to determine the optimal timing for incorporating organic trace minerals and reducing osteomyelitis lameness in broilers, utilizing the aerosol transmission model. The key finding was that adding 0.15% Availa^®^ ZMC to the broilers’ diet during the initial four weeks of growth proved the most effective timing to protect broilers against BCO lameness incidents. This approach surpassed both continuous supplementation and administration in the last four weeks in terms of providing protection against lameness and reducing the cost of feed additives. Further research is necessary to investigate the limitations of applying 0.15% Availa^®^ ZMC or other feed additives during the initial two weeks of production. This approach could contribute to a more efficient and targeted application of additives, potentially yielding enhanced overall health and performance of broiler chickens and reducing the cost of feed additives in the poultry industry.

## Figures and Tables

**Table 1:** Description of the treatment designations in this study.

Dietary Treatment	Description
Diet A in wire floor	BCO source group fed for 56 d
Diet A—Basal diet	Negative control diet fed for 56 d
Diet B—15% Availa^®^ ZMC ^1^	Feeding for 56 d
Diet C—Late feeding	Diet A on 1–28 d switched to Diet B on 29–56 d
Diet D—Early feeding	Diet B on 1–28 d switched to Diet A on 29–56 d

Availa^®^ ZMC, provided by Zinpro Corporation, Eden Prairie, MN, USA, Availa^®^ ZMC diet ^1^, contained 60 mg Zn/kg, 60 mg Mn/kg, and 10.5 mg Cu/kg in the form of mineral amino acid complex.

**Table 2:** Comparison of the cumulative lameness percentages across all treatments in the last three weeks on days 42, 49, and 56.

Day	BCO Source in Wire	Diet A—NC	Diet B	Diet C	Diet D
42	36.00 ^a^	19.00 ^b^	7.00 ^c^	14.00 ^b^	5.00 ^c^
49	65.00 ^d^	38.00 ^e^	19.00 ^f^	40.00 ^e^	17.00 ^f^
56	83.00 ^g^	76.00 ^h^	37.00 ^j^	74.00 ^h^	45.00 ^i^

Diet A: basal diet for 56 days, Diet B: 15% Availa^®^ ZMC for 56 days, Diet C: Diet A on the first four weeks (1–28 d) switched to Diet B on the last four weeks (29–56 d), Diet D: Diet B on the first four weeks (1–28 d) switched to Diet A on the last four weeks (29–56 d). ^a–j^ Values with distinct superscripts in the same category are significantly different at *p* < 0.05.

## Data Availability

Dataset used and/or analyzed in the study is available from the corresponding author upon request (A.A.K.A.).

## Appendix A

**Table A1:** Experimental diets used for additions of Availa^®^ ZMC.

Ingredient Name	Starter Diet (%)		Finisher Diet (%)	
	Diet A ^1^	Diet B ^2^	Diet A ^1^	Diet B ^2^
Corn, ground, 8%	57.12	57.12	64.555	64.55
Soybean meal, 48%	36.76	36.76	29.15	29.15
Poultry oil	2.50	2.50	3.17	3.17
DL-Met	0.34	0.34	0.31	0.31
L-Lys HCl	0.20	0.20	0.21	0.21
L-Thr	0.11	0.11	0.09	0.09
Limestone	1.09	1.09	0.99	0.99
Dicalcium phosphate	1.04	1.04	0.72	0.72
NaCl	0.41	0.41	0.42	0.42
Na bicarbonate	0.08	0.08	0.07	0.07
Phytase ^3^	0.03	0.03	0.03	0.03
Choline chloride, 60%	0.05	0.05	0.05	0.05
Vitamin premix ^4^	0.06	0.06	0.03	0.03
Salinamycin Na ^5^	0.05	0.05	0.05	0.05
Mineral premix ^6^	0.10	0.04	0.10	0.04
Availa^®^ ZMC ^7^	-	0.15	-	0.15
Inert Filler, sand	0.07	-	0.07	-
Total	100.00	100.00	100.00	100.00
Calculated Composition, % unless otherwise noted ^8^				
ME, kcal/kg	3050	3050	3175	3175
CP (%)	22.34	22.34	19.21	19.21
Lys, % digestible	1.22	1.22	1.05	1.05
Met + Cys, % digestible	0.92	0.92	0.82	0.82
Thr, % digestible	0.82	0.82	0.69	0.69
Ca, % total	0.90	0.90	0.76	0.76
P, % available	0.45	0.45	0.38	0.38
Na, % total	0.21	0.21	0.21	0.21
Zn, mg/kg of diet total	100	100	100	100
Mn, mg/kg of diet total	100	100	100	100
Cu, mg/kg of diet total	10	11	10	11

^1^ Control diet. ^2^ Availa^®^ ZMC Diet; ^3^ Optiphos 2000, Huvepharma Inc., Peachtree City, GA, USA. ^4^ The vitamin premix involved (per kg of diet): vitamin A, 3810 IU; vitamin D3, 2722 ICU; vitamin E, 27 IU; vitamin B12 0.001 mg; mendadione, 0.74 mg; riboflavin, 3.26 mg; d-pantothenic acid, 4.90 mg; niacin, 19.05 mg; folic acid, 0.44 mg; pyridoxine, 1.36 mg; thiamine, 0.76 mg. Finisher feed vitamins were reduced 50% from the former levels. ^5^ Salinomycin sodium, Bio-Cox, Huvepharma Inc., Peachtree City, GA. ^6^ The mineral premix involved (per kg of diet when fed at 0.10% of diet): calcium, 56 mg; manganese, 100 mg; magnesium, 27 mg; zinc, 100 mg; iron, 50.0 mg; copper, 10.0 mg; iodine, 1.0 mg. ^7^ Availa ZMC^®^, a mix of Zn, Mn, and Cu amino acid complexes. This mix contains the following rates of each mineral: Zn 4.0%, Mn 4.0%, Cu 0.7% bound into 1 to 1 amino acid ligand. Amino acid contained adds up to 25.1% crude protein. Availa-ZMC, manufactured by Zinpro Corporation, Eden Prairie, MN, USA. ^8^ Zn, Mn, and Cu were analyzed using EPC, with each in the starter and finisher periods. The average total analyzed amounts of Zn, Mn, and Cu fed to the birds were 115, 97, and 16 mg, respectively.

**Table A2:** Average lame and ending average body weights of five clinically healthy birds from each treatment on day 56.

Treatments	Lame Count (wk)	BW (kg) ^2^
	Avg ^1^	Avg ^1^
Diet A—BCO challenge source	43.0 ^a^ ± 2.0	4.08 ^a^ ± 0.25
Diet A—Negative control	39.0 ^a^ ± 1.0	4.64 ^b^ ± 0.29
Diet B	20.2 ^b^ ± 1.7	4.90 ^c^ ± 0.15
Diet C	37.6 ^a^ ± 0.9	4.88 ^c^ ± 0.08
Diet D	23.3 ^b^ ± 0.7	4.94 ^c^ ± 0.17

^a–c^ Values within an experiment that are significantly different (*p* < 0.05) have different superscripts. Diet A: basal diet for 56 days, Diet B: 15% Availa^®^ ZMC for 56 days, Diet C: Diet A on the first four weeks (1–28 d) switched to Diet B on the last four weeks (29–56 d), Diet D: Diet B on the first four weeks (1–28 d) switched to Diet A on the last four weeks (29–56 d). ^1^ Average (Avg) ± SEM, ^2^ BW for five apparently healthy birds at the end of the experiment (day 56).
